# Echocardiographic Parameters as Predictors of In-Hospital Mortality in Patients with Acute Coronary Syndrome Undergoing Percutaneous Coronary Intervention

**DOI:** 10.1155/2014/818365

**Published:** 2014-03-17

**Authors:** Miroslava Sladojevic, Srdjan Sladojevic, Dubravko Culibrk, Snezana Tadic, Robert Jung

**Affiliations:** ^1^Institute of Cardiovascular Diseases Vojvodina, Put Doktora Goldmana 4, 21208 Sremska Kamenica, Serbia; ^2^Department for Telecommunications and Signal Processing, Faculty of Technical Sciences, University of Novi Sad, Trg Dositeja Obradovica 6, 21000 Novi Sad, Serbia; ^3^Department for Industrial Engineering and Management, Faculty of Technical Sciences, University of Novi Sad, Trg Dositeja Obradovica 6, 21000 Novi Sad, Serbia

## Abstract

Different ways have been used to stratify risk in acute coronary syndrome (ACS) patients. The aim of the study was to examine the usefulness of echocardiographic parameters as predictors of in-hospital outcome in patients with ACS after percutaneous coronary intervention (PCI). A data of 2030 patients with diagnosis of ACS hospitalized from December 2008 to December 2011 was used to develop a risk model based on echocardiographic parameters using the binary logistic regression. This model was independently evaluated in validation cohort prospectively (954 patients admitted during 2012). In-hospital mortality in derivation cohort was 7.73%, and 6.28% in validation cohort. Developed model has been designed with 4 independent echocardiographic predictors of in-hospital mortality: left ventricular ejection fraction (LVEF RR = 0.892; 95%CI = 0.854–0.932, *P* < 0.0005), aortic leaflet separation diameter (AOvs RR = 0.131; 95%CI = 0.027–0.627, *P* = 0.011), right ventricle diameter (RV RR = 2.675; 95%CI = 1.109–6.448, *P* = 0.028) and right ventricle systolic pressure (RVSP RR = 1.036; 95%CI = 1.000–1.074, *P* = 0.048). Model has good prognostic accuracy (AUROC = 0.84) and it retains good (AUROC = 0.78) when testing on the validation cohort. Risks for in-hospital mortality after PCI in ACS patients using echocardiographic measurements could be accurately predicted in contemporary practice. Incorporation of such developed model should facilitate research, clinical decisions, and optimizing treatment strategy in selected high risk ACS patients.

## 1. Introduction

Despite the modern methods of diagnosis, advances in treatment over the last three decades, and the implementation of measures of primary and secondary prevention, ACS is still a major threat to the health and life of humans.

Nowadays risk assessment is needed to guide triage and key management decisions. For decades scientists are trying to create ideal scores for predicting risk which is simple, fast, and applicable to everyday practice. Although number of risk scores have been developed to predict short and long term outcomes in patients with ACS [1–10], GRACE and TIMI risk scores are the most popular and validated ACS prediction models, recommended by contemporary guidelines [11, 12].

Echocardiography, as one of most important modality in the acute setting, rapidly and widely available, has significant utility in the diagnosis and management, and is also used for risk stratification and prognosis of ACS patients. Estimated risk, based on echocardiographic predictors, is challenging but there has not been a widespread adoption in the contemporary practice, regardless of the potential benefits this could lead to.

The aim of study was to examine the usefulness of echocardiographic parameters as predictors of in-hospital outcome in patients with ACS after PCI.

The rest of the paper is organized as follows.  Section 2 presents methodology.  Section 3 presents baseline characteristics of the patients from derivation cohort and achieved results.  Section 4 is dedicated to the discussion.  Section 5 holds our conclusions, while Section 6 holds the limitations of the study.

## 2. Meterials and Methods

A set of patient-related data is obtained from the hospital information system from Institute of Cardiovascular Diseases of Vojvodina, situated in Sremska Kamenica, Serbia, Europe.

### 2.1. Study Population

A total of 2030 patients (aged 61.29 ± 11.70 years) hospitalized for ACS and treated with PCI between December 2008 and December 2011 were assigned to a derivation cohort. Validation cohort contained 954 patients (aged 61.54 ± 11.91 years) hospitalized during 2012. All patients were examined by an experienced cardiologist immediately after admission.

### 2.2. Examination

ACS is defined as “any group of clinical symptoms compatible with acute myocardial ischemia”, which includes unstable angina (UA) and myocardial infarction (MI), with or without ST-segment elevation according to American Heart Association [13]. All patients were undergoing an invasive strategy (primary PCI for STEMI/urgent PCI for NSTEMI and UA), within two hours of admission to hospital. Coronary stenting directly, or followed by balloon angioplasty, was performed where eligible. After the procedure, patients were followed in the intensive coronary unit until stabilization.

The echocardiography examination was performed by a Vivid 7 (GE Medical Systems, Horten, Norway) with a phased-array 3.5-MHz transducer. All physicians who applied echocardiography routinely in the Institute were involved. A complete M-mode, two-dimensional and Doppler technique (pulsed, continuous, color and tissue Doppler) examination was performed in all patients during hospitalization, according to the recommendations of the American Society of Echocardiography [14]. Patients were placed in the left lateral decubitus position, and standard parasternal and apical views were obtained after calculating the mean value of three consecutive measurements. LVEF was calculated using a Simpson biplane method of discs [15]. Pulsed and continuous Doppler curves of blood flow were assessed by the apical 4-chamber view [16]. Transmitral pulsed-wave Doppler flow velocity was recorded from the apical 4-chamber view. The sample volume was placed at the mitral valve tips with the ultrasound beam aligned with the mitral inflow, and peak E, A-wave velocities, and E-wave deceleration time were recorded [17]. Mean values of peak velocities resulted from 5 consecutive cardiac cycles.

The following 45 echocardiographic parameters (EP) were obtained in a study presented: IVSd (intraventricular septal thickness at end-diastole), LVIDs (left ventricular internal dimension at end-systole), LVIDd (left ventricular internal dimension at end-diastole), PLWd (posterior wall thickness at end-diastole), AO (the aortic root diameter), AOvs (aortic leaflet separation diameter), LA (left atrial diameter), RV (right ventricular diameter), EDVLV (end-diastolic volume of the left ventricle), ESVLV (end-systolic volume of left ventricle), LVSV (left ventricular stroke volume), EDVLVI (end-diastolic volume of the left ventricle index), ESVLVI (end-systolic volume of the left ventricle index), LVSVI (left ventricular stroke volume index), LVEF (left ventricular ejection fraction), CO (cardiac output), LAab (left atrium apicobasal diameter), RAab (right atrium apicobasal diameter), LAll (left atrium latero-lateral diameter), RAll (right atrium latero-lateral diameter), LVOTd (left ventricular outflow tract diameter), MADd (diastolic mitral annulus diameter), MAAd (diastolic mitral annular area diameter), AscAO (ascending aorta diameter), DscAO (descending aorta diameter), ArchAO (arch aorta diameter), TrcPA (truncus pulmonalis diameter), RVd (right ventricular diameter-diastole), RVs (right ventricular diameter-systole), AOmaxPG (transaortic maximal gradient), AOmeanPG (transaortic mean gradient), AoVTI (aortic velocity-time integral), MVmaxPG (transmitral maximal gradient), MVmeanPG (transmitral mean gradient), MVVTI (mitral velocity-time integral), PVmaxPG (transpulmonic maximal gradient), PVmeanPG (transpulmonic mean gradient), PAVTI (pulmonary artery velocity time integral), AVA (aortic valve area), MVA (mitral valve area), RVSP (right ventricular systolic pressure), Emv (mitral velocity E wave), Amv (mitral velocity A wave), and Emv/Amv, MV Dct (mitral valve deceleration time).

Statistical analysis was performed using SPSS Version 17. Continuous variables were presented as mean ± SD or median (25th percentile–75th percentile). Comparisons between groups were analyzed by unpaired *t*-test or Mann-Whitney test. To identify predictors of intrahospital mortality, the univariate binary logistic regression was used. Including all the variables with *P* < 0.05 the multivariate binary logistic regression gave the model for intrahospital mortality. Receiver operating characteristic (ROC) curve was generated and the area under the curve was calculated. This method was used to investigate the prognostic value of obtained model. Sensitivity and specificity for optimal cutoff were calculated. Differences were considered significant at *P* < 0.05.

## 3. Results

### 3.1. Baseline Characteristics of Patients from Derivation Cohort

The age distribution of 2030 patients belonging to derivation cohort is shown in Table 1. In order for better presentation of derivation cohort population, biochemical analysis of blood parameters on admission is given in Table 2.

As Table 1 shows, the highest percentage (13.3%) of patients in derivation cohort were in the age group of 55.7 to 59.0 years, while younger than 35.9 and older than 85.4 years represent 1.43% patients.

As Table 2 presents, patients with an adverse outcome (exitus letalis) tended to have lower values of Red blood cells, Hemoglobin, Leukocytes, Blood sugar, Creatine phosphokinase, Creatine phosphokinase—MB compared with those who survived. Patients with an adverse outcome tended to have higher value of Urea, Creatinine, Acidum uricum, Bilirubin, Ca++, International normalized ratio, and more frequently positive Troponin, while Fibrinogen tended to have lower value compared with those who did not have an adverse outcome. Sedimentation, Triglycerides, Total cholesterol, Alanine aminotransferase, Lactate dehydrogenase, C-reactive protein, Na^+^, K^+,^ and Total protein did not show significant difference between patient who survived and those who did not.

### 3.2. Echocardiographic Parameters

Each patient from derivation cohort was initially described with 45 echocardiographic parameters (Table 3) which are used for model generation.

As Table 3 shows, patients with an adverse outcome (exitus letalis) tended to have higher values of HR (bat/min.), LVIDs (cm), LA (cm), ESVLV (mL), ESVLVI (mL/m^2^), LAII (cm), RAab (cm), RAII (cm), MAAd (cm), MVmeanPG (mmHg), Emv (cm/sec.), Emv/Amv., RVs (cm), and RVSP (mmHg) compared with patients who survived. Survivors tended to have higher values of AOvs (cm), SVLV (mL), LVEF (%), LVOTD (cm), AOmaxPG (mmHg), AOVTI (cm), AVA (cm^2^), MVVTI (cm), PAmaxPG (mmHg), PAmeanPG (mmHg), PAVTI (cm) compared to those with an adverse outcome. Body weight (kg), Body height (cm), Body Surface Area (m2), LVIDd (cm), IVSd (cm), PLWd (cm), RV (cm), AO (cm), EDVLV (mL), EDVLVI (mL/m2), LVSVI (mL/m2), CO (L/min.), LAab (cm), AOmeanPG (mmHg), AscAO (cm), DscAO (cm), ArchAO (cm), MADd (cm), MVA (cm2), MVmaxPG (mmHg), Amv (cm/sec.), MV Dct. (msec.), RVd (cm), and TrcPA (cm) did not show significant difference between patients who died compared to survivors.

### 3.3. Resulting Model

Using 19 echocardiographic parameters selected by univariate binary logistic regression, the multivariate binary logistic regression gave the model of 4 independent predictors of intrahospital mortality (Table 4).

Consider the following:

(1) tval=2.058−2.03×AOvs+0.984×RV−0.114×LVEF+0.036×RVSPP=exp⁡(tval)(1+exp⁡(tval)).

Developed mathematical model presented in (1) achieved AUROC parameter of 0.840 on derivation cohort. It was tested prospectively on new 954 patients (the validation cohort). It retained good prognostic accuracy (AUROC 0.78). Results are presented in Table 5.

**Figure 1:**
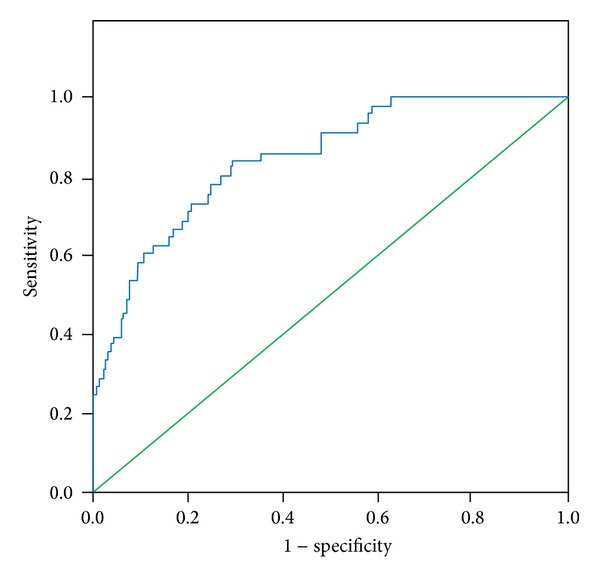
ROC curve achieved on derivation cohort.

**Figure 2:**
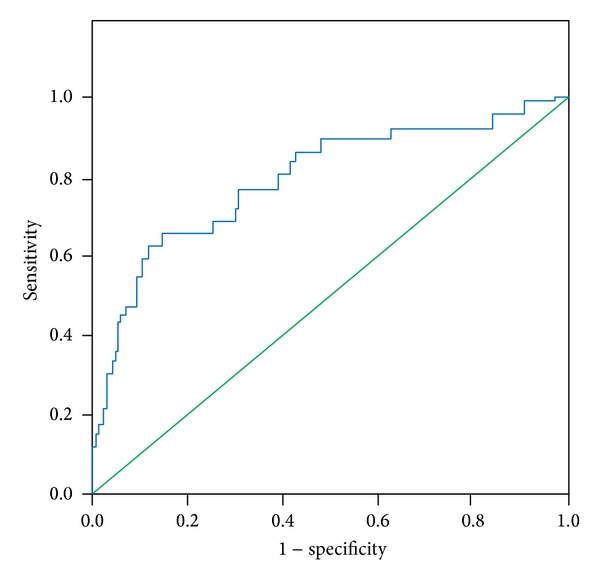
ROC curve achieved on validation cohort.

ROC curves achieved on derivation and validation cohorts are presented in Figures 1 and 2, respectively.

## 4. Discussion

Risk scores are useful tools for the assessment of risk in ACS patients and allow accurate estimations of ischemic and bleeding risk for individual patients. This information is now increasingly used for estimating the patient's risks with the expected benefits and risks associated with available therapies, hence facilitating individual tailoring of treatments.

Nowadays most of predictive models were selected as independent risk factors: age, heart failure, ST segment deviation, and elevated cardiac biomarkers, while a variety of other factors are used in individual models. The most popular risk scoring systems in clinical practice TIMI [11] and the GRACE risk scores [1, 6] have not been optimized for patients undergoing PCI.

In coronary care units, echocardiography as a noninvasive and inexpensive method in comparison with other established methods has significant utility in the diagnosis and management of ACS patients. Echocardiography also has a crucial role in the daily work of clinicians in assessing the risk and prognosis after a coronary incident by evaluating systolic and diastolic left ventricular function. Except ejection fraction others echocardiographic parameters rarely appear as predictors in contemporary risk scores.

Presented study showed that four independent echocardiographic predictor factors have influence on in-hospital mortality: LVEF—left ventricular ejection fraction (*P* < 0.0005), AOvs—aortic leaflet separation diameter (*P* = 0.011), RV—right ventricle diameter (*P* = 0.028), and RVSP—right ventricle systolic pressure (*P* = 0.048).

Left ventricle ejection fraction, the main indicator of left ventricular systolic function, is already known as key prognostic factor of mortality described in many studies, risk scores, and registries like Michigan Risk Score [18], New York Risk Score [19], and Mayo Clinic registry [20]. Lavine and Schwammenthal et al. also reported a LVEF ≤ 0.40 as a powerful and independent predictor of adverse outcome one month after acute MI [21, 22].

AOvs parameter, selected as prognostic factor of mortality in the study, tends to predict adverse outcome with its lower values. It could be explained based on the fact that patients with previous aortic stenosis (smaller AVA, increased AOVTI, and therefore lower AOvs) have a worse prognosis compared to patients without structural disease of the aortic valve in presence of ACS [23]. On the other side, it has been described that patients with ACS, who have increased end-diastolic filling pressure of the left ventricle could lead to early closure of aortic valve. If left ventricular stroke volume is decreased, reduction in flow in late systole may occur, which induce a rounded appearance of aortic valve in late systole [24]. It is already described that increased end-diastolic pressure of the left ventricle, which particularly reflects in the disturbance of left ventricular diastolic function, tends to be associated with large infarctions, but a restrictive pattern is also independently associated with adverse outcome [25].

Pathophysiological explanation for RV and RVSP as predictors of mortality in patients with ACS could be interpreted in many ways. ACS leading to anterograde, LV failure, can have further influence on the pulmonary microcirculation (increased Pulmonary Capillary Wedge Pressure), and then retrograde may be influenced on the increase in RVSP. In study presented RVSP was significantly higher in patients who died. Another explanation is STEMI of the right ventricle, which leads to dilatation of the RV and consecutive increase in RVSP. Although we and others have shown that the presence of RV dysfunction immediately after an MI was an important prognostic factor [26–29], few studies have addressed the prognostic risk associated with late assessment of RV function after an MI [30].

## 5. Conclusion

Goal was to create a model with echocardiographic parameters as predictors which would be easy to use in everyday work, in all institutions, without limitations of preinstalled software of echocardiographic equipment.

The developed echocardiographic model might prove to be very helpful in the decision-making process and optimizing treatment strategy in selected high risk patients presenting with ACS after invasive strategy. It is suitable for expert interpretation, yet relatively simple as it contains only 4 echocardiographic parameters as predictors. Although presented study has been done on a large number of patients and it faithfully illustrates the actual state of patient population of the region where it was developed, authors believe it may find their role in the daily work of clinicians worldwide.

This is still unexplored area and further investigations are needed. For future work it would be interesting to deploy Data Mining techniques including machine learning algorithms in order to find new relationships in the cohorts and extract new useful knowledge.

## 6. Limitation of the Study

The study presented has some limitations. Lack of this study is that some parameters were missing in derivation cohort, like precise parameters of diastolic function, strain rate velocities, myocardial contrast echocardiography, and so forth. Achieved accuracy could be better, taking into account missing parameter and missing values for existing parameters.

Finally, we developed the model for in-hospital mortality, but fatal outcome cannot be excluded after discharge, so the follow-up of this study would be needed.

## Figures and Tables

**Table 1:** Age distribution of patients from derivation cohort.

Age of patient	Number of patients	% of dataset
26.0–29.3	4	0.197
29.3–32.6	3	0.148
32.6–35.9	14	0.690
35.9–39.2	31	1.527
39.2–42.5	62	3.695
42.5–45.8	75	7.340
45.8–49.1	149	7.340
49.1–52.4	137	6.749
52.4–55.7	188	9.261
55.7–59.0	270	13.300
59.0–62.3	205	10.098
62.3–65.6	145	7.143
65.6–68.9	116	5.714
68.9–72.2	209	10.295
72.2–75.5	151	7.438
75.5–78.8	128	6.305
78.8–82.1	104	5.123
82.1–85.4	31	1.527
85.4–88.7	3	0.148
88.7–92.0	5	0.246

**Table 2:** Biochemical analysis of blood parameters on admission in a derivation cohort.

Biochemical analysis of blood parameters on admission	Total (*n* = 2030, 100%)	Exitus letalis = yes (*n* = 157, 7.73%)	Exitus letalis = no (*n* = 1873, 92.27%)	*P*
Sedimentation (mm/h) median (25–75) percentile	20.0 (10.0–37.0)	10.0 (10.0–37.5)	20.0 (10.0–40.5)	0.274

Red blood cells (×10^12^/L) mean ± SD	4.72 ± 0.58	4.61 ± 0.69	4.73 ± 0.57	0.036

Hemoglobin (g/L) median (25–75) percentile	142.0 (131.0–152.0)	109.0 (106.5–141.5)	139.0 (125.5–150.5)	<0.0005

Leukocytes (×10^9^/L) median (25–75) percentile	10.40 (8.40–12.80)	7.80 (6.55–9.30)	9.50 (7.60–12.55)	<0.0005

Triglycerides (mmol/L) median (25–75) percentile	1.80 (1.30–2.50)	0.80 (0.75–0.95)	1.70 (1.10–2.10)	0.536

Total cholesterol (mmol/L) mean ± SD	5.85 ± 1.45	5.69 ± 1.62	5.87 ± 1.43	0.179

Blood sugar (mmol/L) median (25–75) percentile	8.00 (6.30–10.60)	7.90 (7.30–9.75)	7.90 (6.55–11.15)	<0.0005

Troponin positive (%)	51.87	63.69	50.99	<0.0005

Creatine phosphokinase (mmol/L) median (25–75) percentile	628.0 (166.5–1933.0)	244.0 (144.0–545.0)	811.0 (222.0–1704.0)	<0.0005

Creatine phosphokinase-MB (mmol/L) median (25–75) percentile	66.0 (28.0–194.0)	41.0 (31.0–70.5)	84.0 (33.0–190.0)	<0.0005

Bilirubin (*μ*mol/L) median (25–75) percentile	13.00 (10.00–17.00)	16.00 (15.00–33.50)	12.00 (10.50–18.00)	<0.0005

Aspartate aminotransferase (U/L) median (25–75) percentile	42.00 (24.00–120.00)	45.00 (40.50–54.00)	35.00 (25.00–78.50)	0.150

Alanine aminotransferase (U/L) median (25–75) percentile	29.00 (20.00–45.50)	45.00 (42.00–71.50)	28.00 (19.50–44.50)	0.639

Lactate dehydrogenase (U/L) median (25–75) percentile	437.00 (31.75–781.25)	550.00 (426.00–642.50)	380.00 (295.00–537.50)	0.410

Urea (mmol/L) median (25–75) percentile	6.40 (5.10–8.20)	14.70 (9.75–20.10)	6.40 (5.00–8.20)	<0.0005

Creatinine (*μ*mol/L) median (25–75) percentile	93.0 (81.0–108.0)	142.0 (115.0–196.5)	95.0 (79.5–107.5)	<0.0005

C-reactive protein (mg/L) median (25–75) percentile	4.80 (2.10–17.45)	16.50 (10.45–87.05)	3.30 (2.15–10.75)	0.070

Fibrinogen (g/L) median (25–75) percentile	3.70 (3.00–4.60)	3.00 (2.45–4.80)	4.00 (3.10–5.30)	0.001

Quick (%) median (25–75) percentile	86.50 (76.00–97.00)	71.00 (51.50–74.50)	87.00 (75.50–100.00)	<0.0005

International normalized ratio median (25–75) percentile	1.10 (1.00–1.20)	1.30 (1.25–1.85)	1.10 (1.00–1.20)	<0.0005

Na^+^ (mmol/L) median (25–75) percentile	144.0 (141.0–147.0)	143.0 (140.5–144.0)	143.0 (139.0–145.0)	0.835

K^+^ (mmol/L) median (25–75) percentile	4.40 (4.10–4.70)	4.00 (3.90–4.20)	4.40 (4.20–4.75)	0.076

Ca^++^ (mmol/L) median (25–75) percentile	1.210 (1.170–1.250)	1.140 (1.130–1.155)	1.210 (1.180–1.255)	0.009

Acidum uricum (*μ*mol/L) median (25–75) percentile	321.0 (262.0–387.0)	447.0 (372.5–596.5)	330.0 (266.0–390.5)	<0.0005

Total protein (g/L) median (25–75) percentile	76.0 (71.0–80.0)	66.0 (61.0–73.0)	73.0 (69.5–78.0)	0.687

**Table 3:** Echocardiographic parameters in derivation cohort.

Echocardiographic parameter (EP)	Total (*n* = 2030, 100%)	Exitus letalis = yes (*n* = 157, 7.73%)	Exitus letalis = no (*n* = 1873, 92.27%)	*P*
TT (kg) median (25–75) percentile	82.00 (73.00–93.00)	78.50 (65.00–80.00)	82.00 (73.00–93.00)	0.212

TV (cm) median (25–75) percentile	173.00 (165.00–180.00)	169.00 (167.25–182.00)	173.00 (165.00–180.00)	0.835

HR (bat/min.) mean ± SD	80.15 ± 22.90	109.80 ± 19.63	78.22 ± 21.84	0.002

BSA (kg/m^2^) mean ± SD	1.96 ± 0.21	1.87 ± 0.15	1.95 ± 0.21	0.432

LVIDs (cm) median (25–75) percentile	3.30 (2.90–3.70)	3.60 (3.05–4.00)	3.30 (2.90–3.70)	0.000

LVIDd (cm) median (25–75) percentile	5.00 (4.70–5.40)	5.00 (4.68–5.50)	5.00 (4.70–5.40)	0.617

IVSd (cm) mean ± SD	1.18 ± 0.13	1.17 ± 0.14	1.18 ± 0.13	0.531

PLWd (cm) median (25–75) percentile	1.20 (1.10–1.30)	1.15 (1.00–1.20)	1.20 (1.10–1.30)	0.087

LA (cm) median (25–75) percentile	3.70 (3.40–3.90)	3.80 (3.40–4.30)	3.70 (3.40–3.90)	0.043

RV (cm) median (25–75) percentile	2.20 (2.00–2.40)	2.20 (2.00–2.53)	2.00 (1.80–2.00)	0.071

AO (cm) median (25–75) percentile	3.40 (3.20–3.60)	3.30 (3.10–3.50)	3.40 (3.20–3.60)	0.307

AOvs (cm) median (25–75) percentile	2.00 (1.80–2.00)	1.80 (1.60–2.00)	2.00 (1.80–2.00)	<0.0005

EDVLV (mL) median (25–75) percentile	99.00 (85.00–121.00)	109 (80.50–133.50)	99.00 (85–120.88)	0.379

EDVLVI (mL/m^2^) mean ± SD	51.28 ± 15.29	60.53 ± 25.50	51.15 ± 15.20	0.391

ESVLV (mL) median (25–75) percentile	47.00 (38.00–62.00)	65 (46.25–85.25)	47 (37–61)	<0.0005

ESVLVI (mL/m^2^) median (25–75) percentile	23.56 (18.37–31.96)	48.21 (30.85–107.84)	23.41 (18.36–31.88)	0.021

SVLV (mL) median (25–75) percentile	50.00 (43.00–60.00)	40 (30–48.50)	51 (43–60)	<0.0005

SVLVI (mL/m^2^) mean ± SD	26.77 ± 6.50	22.02 ± 9.48	26.81 ± 6.46	0.204

LVEF (%) median (25–75) percentile	52.00 (45.00–57.00)	38 (28–47)	52 (45–58)	<0.0005

CO (L/min.) mean ± SD	3.77 ± 1.12	3.66 ± 1.12	3.78 ± 1.13	0.865

LVOTD (cm) median (25–75) percentile	2.00 (1.90–2.10)	1.90 (1.70–2.00)	2.00 (1.90–2.10)	0.006

LAab (cm) median (25–75) percentile	5.40 (5.00–5.70)	5.55 (4.90–6.20)	5.40 (5.00–5.70)	0.125

LAII (cm) median (25–75) percentile	3.90 (3.60–4.30)	4.30 (3.90–4.70)	3.90 (3.60–4.30)	<0.0005

RAab (cm) median (25–75) percentile	4.80 (4.40–5.20)	5.05 (4.57–5.70)	4.80 (4.40–5.10)	0.008

RAII (cm) median (25–75) percentile	3.40 (3.10–3.70)	3.70 (3.30–4.35)	3.40 (3.10–3.70)	<0.0005

AOmaxPG (mmHg) median (25–75) percentile	6.90 (5.20–9.30)	5.9 (4.4–8.8)	6.9 (5.20–9.30)	0.008

AOmeanPG (mmHg) median (25–75) percentile	3.60 (2.70–4.90)	3.10 (2.35–5.20)	3.60 (2.70–4.90)	0.086

AOVTI (cm) median (25–75) percentile	25.10 (21.00–30.00)	22.00 (15.00–29.50)	25.60 (21.20–30.00)	0.001

AVA (cm^2^) mean ± SD	2.23 ± 0.67	1.63 ± 0.61	2.26 ± 0.66	<0.0005

AscAO (cm) median (25–75) percentile	3.70 (3.40–3.90)	3.70 (3.50–3.90)	3.70 (3.40–3.90)	0.680

DscAO (cm) mean ± SD	2.16 ± 0.35	1.85 ± 0.21	2.18 ± 0.35	0.068

ArchAO (cm) mean ± SD	2.84 ± 0.31	2.71 ± 0.29	2.85 ± 0.31	0.218

MADd (cm) median (25–75) percentile	3.10 (2.90–3.30)	3.20 (3.00–3.40)	3.10 (2.90–3.30)	0.078

MAAd (cm) median (25–75) percentile	7.54 (6.60–8.55)	8.04 (7.06–9.07)	7.54 (6.60–8.55)	0.043

MVA (cm^2^) mean ± SD	5.08 ± 1.15	4.46 ± 1.18	5.10 ± 1.14	0.056

MVmaxPG (mmHg) median (25–75) percentile	3.60 (2.70–4.80)	3.80 (2.60–6.05)	3.60 (2.70–4.80)	0.253

MVmeanPG (mmHg) median (25–75) percentile	1.50 (1.02–2.00)	1.80 (1.10–2.50)	1.50 (1.00–2.00)	0.044

MVVTI (cm) median (25–75) percentile	23.00 (18.78–28.00)	19.00 (14.75–26.00)	23.50 (19.00–28.00)	<0.0005

Emv (cm/sec.) median (25–75) percentile	0.80 (0.60–0.90)	1.00 (0.70–1.05)	0.70 (0.50–0.90)	0.017

Amv (cm/sec.) median (25–75) percentile	0.70 (0.60–0.90)	0.55 (0.38–0.83)	0.70 (0.60–0.90)	0.109

Emv/Amv median (25–75) percentile	1.00 (0.70–1.30)	1.55 (1.17–2.18)	0.95 (0.70–1.30)	0.028

MV Dct. (msec.) mean ± SD	152.53 ± 73.13	119.37 ± 104.84	153.38 ± 72.58	0.429

RVd (cm) mean ± SD	4.39 ± 0.91	4.63 ± 1.19	4.26 ± 0.76	0.437

RVs (cm) mean ± SD	3.54 ± 0.91	4.12 ± 0.63	3.19 ± 0.91	0.045

RVSP (mmHg) median (25–75) percentile	33.00 (26.00–39.00)	38.00 (31.00–45.00)	33.00 (26.00–39.00)	<0.0005

TrcPA (cm) median (25–75) percentile	2.10 (1.90–2.20)	2.00 (1.90–2.15)	2.10 (1.90–2.20)	0.367

PAmaxPG (mmHg) median (25–75) percentile	3.50 (2.70–4.80)	2.80 (1.73–4.45)	3.50 (2.70–4.80)	0.042

PAmeanPG (mmHg) median (25–75) percentile	1.70 (1.21–2.40)	1.15 (0.63–2.15)	1.80 (1.30–2.40)	0.010

PAVTI (cm) median (25–75) percentile	18.55 (15.93–22.00)	11.60 (7.25–18.23)	18.95 (16.00–22.00)	<0.0005

**Table 4:** Binary logistic regression on derivation cohort.

Echocardiography parameter	Univariante analysis		Multivariante analysis	
	RR 95% CI for RR	*P* value	RR 95% CI for RR	*P*-value
HR (bat/min.)	1.56 (1.013–1.102)	0.011		
LVIDs (cm)	1.919 (1.406–2.618)	<0.0005		
LA (cm)	2.035 (1.252–3.308)	0.004		
AOvs (cm)	**0.055 (0.023–0.132)**	**<0.0005**	**0.131 (0.027–0.627)**	**0.011**
RV (cm)	**2.056 (1.157–3.654)**	**0.014**	**2.675 (1.109–6.448)**	**0.028**
ESVLV (mL)	1.019 (1.013–1.026)	<0.0005		
ESVLVI (mL/m^2^)	1.068 (1.027–1.110)	0.001		
SVLV (mL)	0.923 (0.903–0.942)	<0.0005		
LVEF (%)	**0.888 (0.869–0.908)**	**<0.0005**	**0.892 (0.854–0.932)**	**<0.0005**
LVOTD (cm)	0.019 (0.001–0.255)	0.003		
LAII (cm)	2.135 (1.135–3.367)	0.001		
RAab (cm)	1.984 (1.281–3.074)	0.002		
RAII (cm)	3.541 (2.156–5.815)	<0.0005		
MVVTI (cm)	0.908 (0.868–0.950)	<0.0005		
Emv (cm/sec.)	50.992 (2.492–1043.99)	0.011		
RVSP (mmHg)	**1.057 (1.034–1.081)**	**<0.0005**	**1.036 (1.000–1.074)**	**0.048**
PAmaxPG (mmHg)	0.684 (0.478–0.979)	0.038		
PAmeanPG (mmHg)	0.314 (0.133–0.742)	0.008		
PAVTI (cm)	0.749 (0.662–0.846)	<0.0005		

**Table 5:** Results of the model achieved by multivariate binary logistic regression.

	AUROC	*P* value	Cutoff value	Sensitivity	Specificity
Derivation cohort	0.840	<0.0005	4.4	80.0%	77.9%
Validation cohort	0.782	<0.0005	7.64	64.7%	85.8%
